# Myocardial Architecture, Mechanics, and Fibrosis in Congenital Heart Disease

**DOI:** 10.3389/fcvm.2017.00030

**Published:** 2017-05-23

**Authors:** Sarah Ghonim, Inga Voges, Peter D. Gatehouse, Jennifer Keegan, Michael A. Gatzoulis, Philip J. Kilner, Sonya V. Babu-Narayan

**Affiliations:** ^1^Adult Congenital Heart Unit, Royal Brompton Hospital, London, UK; ^2^Cardiovascular Magnetic Resonance Unit, Royal Brompton Hospital, London, UK; ^3^National Heart and Lung Institute, Imperial College, London, UK

**Keywords:** cardiology, congenital heart disease, cardiovascular magnetic resonance imaging, late gadolinium enhancement cardiovascular magnetic resonance, myocardial strain, fibrosis, diffusion tensor imaging

## Abstract

Congenital heart disease (CHD) is the most common category of birth defect, affecting 1% of the population and requiring cardiovascular surgery in the first months of life in many patients. Due to advances in congenital cardiovascular surgery and patient management, most children with CHD now survive into adulthood. However, residual and postoperative defects are common resulting in abnormal hemodynamics, which may interact further with scar formation related to surgical procedures. Cardiovascular magnetic resonance (CMR) has become an important diagnostic imaging modality in the long-term management of CHD patients. It is the gold standard technique to assess ventricular volumes and systolic function. Besides this, advanced CMR techniques allow the acquisition of more detailed information about myocardial architecture, ventricular mechanics, and fibrosis. The left ventricle (LV) and right ventricle have unique myocardial architecture that underpins their mechanics; however, this becomes disorganized under conditions of volume and pressure overload. CMR diffusion tensor imaging is able to interrogate non-invasively the principal alignments of microstructures in the left ventricular wall. Myocardial tissue tagging (displacement encoding using stimulated echoes) and feature tracking are CMR techniques that can be used to examine the deformation and strain of the myocardium in CHD, whereas 3D feature tracking can assess the twisting motion of the LV chamber. Late gadolinium enhancement imaging and more recently T1 mapping can help in detecting fibrotic myocardial changes and evolve our understanding of the pathophysiology of CHD patients. This review not only gives an overview about available or emerging CMR techniques for assessing myocardial mechanics and fibrosis but it also describes their clinical value and how they can be used to detect abnormalities in myocardial architecture and mechanics in CHD patients.

## Ventricular Architecture and Mechanics

### Architecture of the Left Ventricle (LV)

The normal myo-architecture of the heart differs between the LV and the right ventricle (RV). The LV has a thicker compact layer with its myocytes arrayed in varying orientations through its depth, while the more apical parts of the human RV are predominantly trabeculated, with only a thin outermost compact layer.

The LV subepicardial layer contains fibers orientated in a left-handed helical arrangement, which is largely responsible for torsion of the apex relative to the base. The mid-wall layer contains circumferential fibers that generate radial contraction. The subendocardial layer has right-handed helical as well as longitudinal fibers that function in conjunction with the helical subepicardial layer and papillary muscles to generate longitudinal strain (1, 2). The ventricular myocardial fibers are connected and are generally aligned with their neighbors, with only gradual change in the direction of the fibers from layer to layer (3, 4). Because of their opposing helical orientation, myocytes of the subepicardial and subendocardial layers of a given wall region contract almost orthogonally to one another during systole. They and those of the circumferential mid-layer also counter thicken, as they maintain their cell volume. In the compact myocardium, myocytes are also aggregated in micro-laminar arrays known as sheetlets, with intervening cracks or shear layers. These laminar arrays of sheetlets all slope obliquely relative to the local wall tangent plane and change orientation through the cycle, tilting to be more wall perpendicular in systole and more wall parallel in diastole. This reorientation of micro-laminar structures not only accommodates the counter thickening of myocytes in the circumferential layer while those of the endocardial and epicardial layers are contracting but also translates this forced cross-myocyte shortening into enhanced wall thickening (5–7). In the normal myocardium, the LV wall thickens radially by 30–50%, resulting in a normal ejection fraction. Yet, on a cellular level, myocytes shorten only by about 15% thus increasing their mean diameter by about 8% (8, 9). The change in sheetlet orientation in systole is thought to account for most of the wall thickening seen (5, 6, 10). The overall result of the combined twisting, laminar reorientation, and compressive forces is the ejection of the stroke volume from the LV cavity.

It may also be the case that diastolic relaxation of the laminar structures facilitates perfusion, in diastole, of blood through microvessels passing between the sheetlets; however, the papillary muscles and the trabeculars that are particularly prevalent in the RV lack laminar microstructures (11). Their less complex but nevertheless dense local myocardial structure, combined with their relative remoteness from epicardial coronary arteries, may predispose them to ischemia if abnormally loaded, for example, by ventricular volume and/or pressure loading. Regrettably, current methods of myocardial perfusion imaging lack the spatial resolution to confirm this, but it may be surmised that trabecular ischemia is a contributor to the gradual dysfunction of RVs that are hypertrophied due to chronic pressure and volume overload. In a study by Babu-Narayan et al., focal fibrosis as represented by late gadolinium enhancement (LGE) was found in trabeculation remote from surgical sites in patients with repaired tetralogy of Fallot (rToF) (12).

In conditions with pressure or volume overloading, changes in LV myo-architecture have been reported. An increase in the amount of longitudinal fibers was found in the subendocardial layer, and a decreased number of circumferential fibers in the mid-layer were found. Furthermore, a change in orientation of the subendocardial fibers was noted (13, 14).

### Architecture of the RV

The myo-architecture of a normal RV wall is not considered to contain a middle layer of circumferential fibers apart from the right ventricular outflow tract (RVOT) (15). However, in a diseased RV [tetralogy of Fallot (ToF)], a middle circumferential layer was identified (13).

Histological studies have demonstrated disorganization in the RV myocardial architecture in patients with ToF. They have been found to have a more substantial proportion of circumferential fibers in the hypertrophied sub-pulmonary infundibulum (15). A key difference in myo-architecture in ToF is the presence of circumferential fibers in the mid-wall, which is particularly abundant in hypertrophied RV cavities and may be responsible for the reduced compliance (13). These changes in myo-architecture were not only found in adult ToF patients’ post-repair but also in infants before surgery (13).

The RV has a complex geometrical shape. The RV is wrapped around the LV, which allows it to shorten in systole as well as to benefit from ventricle–ventricle interdependence from the LV contraction due to its sharing of common fibers, septum, and pericardial space. The RV subendocardial fibers are shared with the LV subendocardial layer *via* the interventricular septum. Likewise, the RV subepicardial fibers are shared with the LV subepicardial layer (16). Chronic RV pressure or volume overload in congenital heart disease (CHD) such as ToF, conditions with a systemic RV, Ebstein’s anomaly, and atrial septal defects alter the mechanical properties of the interventricular septum whereby it becomes strained and deformed. Consequently, LV function can be affected (17). It has also been reported that on a cellular level abnormal septal mechanics induce a process of apoptosis and dysregulation of the angiogenic factors in the LV wall that can further impair LV function (17).

### The Myocardial Extracellular Space

The myocardial extracellular space is the interstitial tissue that contains the fibro-collagenous material, endomysium. The endomysium acts like a mesh that coordinates the conduction of impulses and transmission of forces and provides a supportive framework. In immediate proximity to cardiac myocytes is the perimysium, which is the thicker connective tissue that transmits shearing forces. Abnormal accumulation and/or change in the quality of the connective tissue increases myocardial stiffness and reduces the compliance of the ventricle (5–7).

Relatively few studies have investigated the significance of collagenous matrix with relation to CHD (18, 19), the most relevant of which was a study that quantified the collagen matrix in 23 heart specimens with univentricular repair of hypoplastic left heart syndrome (HLHS) compared to a control group. Hearts with HLHS had significantly less collagen fibrous matrix in the RV (the systemic ventricle) and LV compared to normal hearts. In addition intrinsic myocardial abnormality could also be found in the hypoplastic LV. Myocardial fibrosis, therefore, could potentially affect long-term function and outcomes of the systemic RV (20).

### Cardiac Diffusion Tensor Imaging (cDTI)

**Figure 1:**
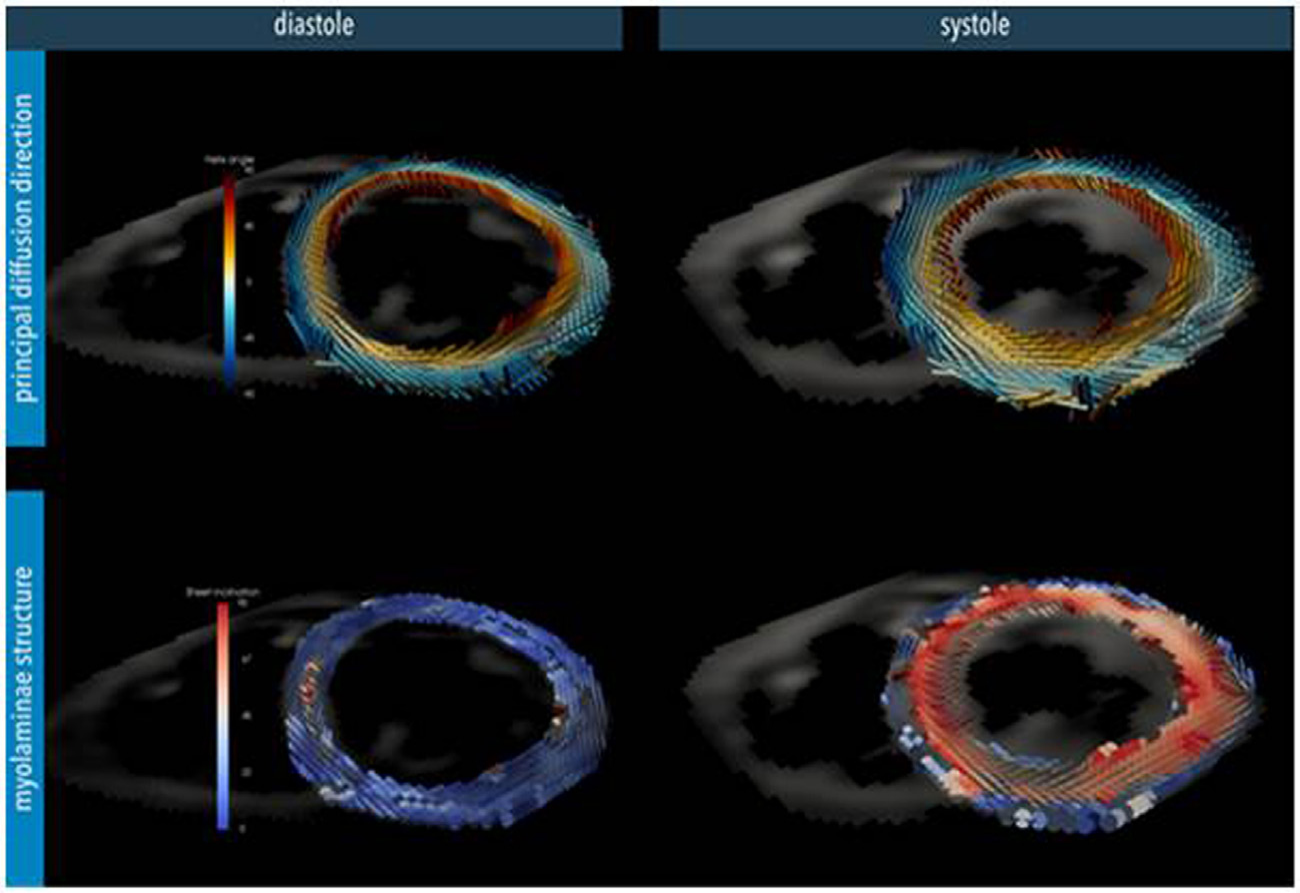
**Myocardial microstructure probed with diffusion tensor imaging in a short-axis slice at peak systole and diastole in a healthy heart**. Top: the principal diffusion direction of water molecules is along the local myocyte long-axis orientation, depicting its helical arrangement. Bottom: additional diffusion directions also hint at the myocardial laminar organization where sheets of myocytes are surrounded by collagen-lined shear layers. These myo-laminae moderate cellular reorganization during cardiac contraction. In the normal heart, myocyte orientation remains similar between systole and diastole, but there is a tilting of the sheets toward a more wall-perpendicular conformation during systole (24). (Image reconstruction below is courtesy of Pedro Ferreira, Senior Physicist, Royal Brompton Hospital.)

Cardiac diffusion tensor imaging has long been utilized in the imaging of the central nervous system. However, more recent developments in cDTI sequences have enabled its use in the non-invasive interrogation of the myo-architecture and fiber orientation. Until recently, cDTI was mainly used to investigate myofiber orientation in *ex vivo* heart tissues due to the problems of imaging beating hearts because of motion artifact. Results of cDTI have been verified with histological findings in a number of studies (21, 22). More recently, sequence developments have enabled *in vivo* use for cDTI and there is increasing evidence that cardiovascular disease processes can be associated with abnormal cDTI parameters (10). Water has the ability to diffuse in all directions; however, microstructural boundaries may limit molecular displacement in certain directions more than others (anisotropic diffusion). In the myocardium, water diffusion is thought to be limited by cell boundaries and their aggregates in sheetlets. Diffusion of water appears to be greatest in directions parallel to myocytes. In cDTI, the direction and the magnitude of diffusion of water molecules are measured for each voxel, which will contain numerous myocytes and sheetlets. After a diffusion gradient is applied, the greatest signal attenuation, i.e., the darkest signal, is obtained when the diffusion gradients are parallel to the length of the myofibers. A diffusion tensor is calculated from measurements of diffusion in six or more directions. A diffusion tensor represents the anisotropic diffusion of water in three dimensions. The principle eigenvector (E1) is therefore the value that represents the direction of water diffusion along the length of the myofiber from which the direction of the myofiber can be inferred. The greatest vector (E2) represents the largest magnitude of diffusion when the myofibers are en face. E3 is the smallest eigenvector. Diffusion tensor imaging (DTI) measures the average of the eigenvectors for each voxel. An *in vivo* sequence has been developed for cDTI using imaging at 3 T. A diffusion weighted stimulated echo acquisition mode (STEAM) sequence is used in conjunction with echo planer imaging, a hybrid single-shot spin echo and gradient echo sequence. This sequence works over two cardiac cycles and assumes that myocardial tissue returns to the same position, at both diffusion encoding times at end systole. Parallel imaging is applied to reduce the length of single-shot imaging in each cardiac cycle. Each 2D slice can be acquired in breath-holds of 18 heart beats duration. Typically eight breath-hold acquisitions are performed per 2D slice and the data averaged to improve signal-to-noise ratio (SNR) (10). Complex postprocessing techniques allow for E1 mapping giving a 3D visual display of the orientation of myofibers in the wall of the LV (Figure 1). Limitations of the cDTI STEAM sequence to date are low SNR, low spatial resolution, and susceptibility to strain artifact (23), and it is dependent on a regular R–R interval.

Cardiac DTI has been applied in some studies to interrogate myocardial fiber orientation in certain disease processes; however, to date, very little has been trialed with respect to CHD. Investigators have studied hypertrophic cardiomyopathy and have identified abnormal orientation of myocardial fibers in diastole that remains in a relatively systolic conformation (10). This was thought to be responsible for the abnormal wall thickness seen in hypertrophic cardiomyopathy rather than replacement fibrosis seen by LGE. cDTI has also been used to study ventricular remodeling postinfarction (25, 26). There are few data for the use of DTI in CHD.

### Ventricular Function Analysis

Cardiovascular magnetic resonance (CMR) has the capability of providing important data on global and regional ventricular function. It is the gold standard in the estimation of ventricular volumes and ejection fraction using a disk-area summation method and in comparison to other modalities as it does not rely on assuming ventricular geometry (27, 28). For global and regional assessment of ventricular function, cine imaging with a balanced steady-state free precession (bSSFP) sequence can provide useful functional information. These ideally are acquired in breath-hold in order to achieve the sharpest delineation between the myocardium and blood pool. Furthermore, bSSFP imaging uses a high flip angle as well as short-echo time and short-repeat time in order to produce images with a high SNR and therefore better contrast between blood and myocardium. Typically, data acquisition is segmented over 10–12 cardiac cycles. The data segment for a given cardiac cycle being acquired is then repeated at multiple time points in the cardiac cycle to create cine phases. The acquired images are repeated on a loop to form cine. Normal flow in the ventricle is represented on these “white-blood” images as high signal whereas turbulence, stenosis, regurgitation, or shunt is represented as low signal. For further assessment of function, using bSSFP images, RV and LV end-diastolic and end-systolic volumes, RV and LV mass, stroke volume, and ejection fraction can be derived.

While acquiring SSFP images in breath-hold, misregistration artifact may arise. This is due the natural variability in the size and depth of breath a patient may take that can cause small changes in the location of the heart each time between acquisitions. Secondly, there are the physiological effects of respiration (e.g., increased venous return) that may affect reproducibility. Four-dimensional, multiphase, steady-state imaging with contrast enhancement is a respiratory gated technique that may overcome breath-holding problems that was developed by Han and colleagues in 2014 to study pediatric patients with complex CHD. The results of this small study showed good correlation of cardiac volumes with conventional 2D cines (29).

## Myocardial Strain

Markers of intrinsic myocardial contractility are investigated with the aim of identifying early myocardial abnormalities that are subclinical and precede ventricular systolic dysfunction. Myocardial strain is a measure of how much myocardial tissue gets larger and thickens (positive strain) or how much it gets smaller or thins (negative strain) in relation to its state in end diastole. It occurs in three different planes in accordance with the orientation of myofibers in the LV subepicardial (helical), middle (circumferential), and subendocardial (longitudinal) layers as described above. Several CMR methods have been developed to interrogate myocardial strain in these planes. Myocardial tagging has been long established as a gold standard technique (30). In this technique, images are acquired in systole and diastole following the disruption of the magnetic field with multiple radio-frequency saturation pulses using sequences such as harmonic phase analysis or spatial modulation of magnetization. Parallel saturation lines (or tags) are superimposed on the myocardium and their deformation with cardiac motion imaged in a cine acquisition. However, while the technique is visually appealing the spatial resolution is limited to the tag spacing and processing time is very long as automatic detection of the tags and their displacement through the cardiac cycle is difficult. This is particularly so in the late phase of the cardiac cycle when the tags have faded. Displacement encoding using stimulated echoes (DENSE) (31) and tissue tracking (two and three dimensional) (32) are newer techniques which in some studies have shown equivalent accuracy (33). These techniques are discussed in depth by Simpson and colleagues in 2012 (34).

### Displacement Encoding Using Stimulated Echoes

Displacement encoding using stimulated echoes is a newer MRI technique that was developed after myocardial tagging. It has a relatively high spatial resolution that can be used to quantify the displacement and strain of the myocardium on a pixel by pixel basis. With this technique, displacement information is directly encoded in to the phase of the sequence for each voxel 2D or 3D image, and a displacement map can be produced. This is used to calculate strains in longitudinal, circumferential, and radial directions in tissue.

The original DENSE sequence produced a displacement-encoded image at a single-time point in the cardiac cycle. Subsequently, cineDENSE incorporated highly efficient data acquisition to produce images throughout the cardiac cycle (33, 35), while fast cine DENSE incorporated parallel imaging and other advanced image processing techniques to improve SNR and spatial and temporal resolution in a reduced acquisition time (36). To date, however, DENSE is still a research tool and has not been used for strain analysis in clinical management of CHD.

### Feature Tracking-CMR (FT-CMR)

Feature tracking-CMR is a technique that is comparable to speckle tracking in echocardiography. Although it is not a direct measure of “true” strain in the myocardium, it is an estimate of endocardial contractility and relaxation properties. The postprocessing technique identifies patterns of features or irregularities in a small window and searches for the same pattern of features in the following images. The measured displacements between the two patterns enable a feature-tracked strain-related index (FTSI) to be calculated. The tissue represented by each voxel in the cine images has their FTSI measured in the longitudinal, radial, and circumferential directions (37).

Longitudinal and circumferential FTSI are derived by tracking tissue components along the length of the myocardium in the long axis. Radial FTSI is estimated from tracking tissue components in the perpendicular direction to the myocardium in the short axis. In a normal heart, there is predominantly a positive radial strain that thickens the LV wall and negative longitudinal and circumferential strains that reduce LV cavity size in systole. Tissue components of interest are preferably selected along the endocardial border rather than the epicardial border as the reduction of the endocardial surface is a better representation of the efficiency of the LV cavity emptying its stroke volume (38).

Rapid acquisition of images is performed using a bSSFP sequence. Current bSSFP images are good at distinguishing blood pool from myocardium with a spatial resolution of 1–2 mm (in-plane). However, with CMR, it is difficult to distinguish features within the compact myocardium due to its homogeneity and large size voxels. Therefore, instead of tracking features within the myocardium, FT-CMR utilizes the endocardial border features that become easily distinguishable by CMR. One limitation of CMR is its limited spatial resolution through-plane (6–10 mm), which means that FT-CMR is unable to track features that move out of plane in subsequent frames in the through-plane direction (38).

3D-FT-CMR is an area of growing interest where all three strain parameters can be measured from a single-3D acquisition. An assumption of shear motion between the layers (circumferential–longitudinal, longitudinal–radial, and circumferential–radial) may be made. The shear motion between the circumferential–longitudinal layers in particular is responsible for LV torsional motion. 3D-FT-CMR is again limited by lower through-plane spatial resolution, and therefore it is not ideal in measuring longitudinal strain in the short-axis LV stacks; however, it has shown comparable results for radial and circumferential strains. 3D-FT-CMR involves longer acquisitions and so a navigator is required for avoiding respiratory motion artifact.

Several studies have compared FT-CMR with myocardial tagging techniques and have demonstrated good agreement in circumferential strain measurements recorded between both techniques in healthy subjects, aortic stenosis, and non-ischemic cardiomyopathy (39–41). FT-CMR has the advantage of not requiring any additional imaging to cine images acquired and has a shorter postprocessing time (42). Interobserver variability has been reported to be at least equivocal to myocardial tagging for circumferential strain measurements. However, FT-CMR-derived longitudinal and radial strain, however, is not as reliably correlated with myocardial tagging (39).

### Clinical Applications of Feature Tracking

Patients with *ToF* commonly have RV dysfunction that can progress through their adult life and several mechanisms for this have been described (please see above). RV dysfunction is in keeping with impaired myocardial strain and intraventricular RV dyssynchrony and interventricular dyssynchrony. Several studies have used FT-CMR to examine strain and its prognostic significance.

It has been reported ToF patients who experience adverse outcomes (death or sustained ventricular tachycardia), all have globally lower RV FTSI indices compared to patients with no adverse outcome. Impaired longitudinal “strain” of both ventricles was strongly associated with adverse clinical outcomes (43). Orwat et al. also recently studied the correlation between FT-CMR derived indices with adverse outcomes. They reported that RV-longitudinal FTSI and LV-circumferential FTSI were predictors of adverse outcomes independent of other known risk factors and suggested that these parameters should be included in the risk assessment process (44). Jing and colleagues did not find a correlation between FT-CMR parameters and RV dilation and RVEF, reporting that they were not independent predictors of developing RV dysfunction and subsequent adverse outcomes (45).

Feature tracking-CMR has been used to investigate RV function following different types of RVOT obstruction (RVOTO) repair. The current move in surgical approach to relieve RV infundibular obstruction is to do perform minimum resection and preserve the pulmonary valve in order to prevent pulmonary valve incompetence (46). The effect of this approach is to leave residual RVOTO. It is thought that the increased RV pressure overload causes hypertrophy and increased contractile function. Several investigators have reported this to be protective on RV remodeling in comparison to a volume overloaded RV from pulmonary regurgitation. Latus et al. used FT-CMR to investigate the underlying mechanism for this and found that residual RVOTO following a more conservative surgical approach, RV circumferential and radial strain were higher than in the control group though RV longitudinal strain did not change (47). Stronger RV–LV interaction and reduced ventricular dyssynchrony were reported (47). LV longitudinal strain, which was reduced in the RVOTO group, an area that still needs further investigation, given LV dysfunction is an adverse prognostic marker for mortality in ToF.

In atrial redirection surgery for *the great arteries (TGA)*, Tutarel et al. also found that circumferential FTSI correlated moderately with RV (systemic) ejection fraction and that there was a negative correlation with QRS duration; weak correlations between FTSI and LVEF were found (48). Velocity vector imaging as part of FT-CMR was used in another study to compare “strain” parameters in patients after atrial redirection surgery for TGA with patients who underwent arterial switch operation (49). Patients after atrial switch operation showed reduced RV ejection fraction (RVEF) and decreased longitudinal and circumferential strain parameters compared to patients with arterial switch operation (49).

The prognostic significance of FT-CMR derived indices were assessed in a small group of adults with *single-ventricle* post-Fontan surgery. Systemic ventricle longitudinal, radial, and circumferential FTSI correlated with NYHA class, peak oxygen uptake on cardiopulmonary exercise testing, and age of complete Fontan surgery (50).

### Ventricular Regional and Global Function for Predicting Outcomes

More sensitive parameters for both regional ventricular function and intrinsic myocardial contractility are sought after in order to identify abnormalities early when patients are asymptomatic prior to the establishment of systolic dysfunction. Often when systolic dysfunction sets in, it is indicative of an advanced stage of the myocardial disease process. In patients with repaired ToF, several CMR parameters of ventricular function have been assessed with their prognostic significance determined. RVOT regional wall motion abnormalities were found to have important prognostic implications. RVOT aneurysms or akinetic RVOT portions are common features related to scar tissue due to transannular patching or previous generous infundibular stenosis resection (12, 51). These akinetic RVOT areas contribute to a larger RV end-diastolic and end-systolic volume (RVEDV and RVESV) hence having a negative impact on the RVEF (12, 52). Delay of conduction is also found within this region leading to prolongation of QRS elongation, representing intraventricular (RV) dyssynchrony (53). Bonello et al. have reported a correlation between the length of the akinetic RVOT wall and the first onset of ventricular arrhythmias (54). Another marker of function is RV volumes. Studies have focused on these to determine preoperative thresholds that predict timing for pulmonary valve replacement surgery that results in normalization of RV volumes in rToF patients (55). As an overall consensus from several studies, indexed RVEDV between 150 and 170 ml (and 158 ml/m^2^ from our center) or indexed RVESV between 80 and 90 ml (and 82 ml/m^2^ from our center) are when pulmonary valve replacement for pulmonary regurgitation should be considered for asymptomatic patients (56). Past this point, it is thought that a degree of permanent RV damage has occurred. Following an optimally timed pulmonary valve replacement, RV volumes improve at CMR (57).

Left ventricle longitudinal impairment was reported to be a predictor of sudden cardiac death (SCD) in patients with repaired ToF (58). The INDICATOR multicentre trial in 2014 investigated 873 adult Fallot patients found that RV hypertrophy that was out of proportion to volume dilation (quantified on CMR by RV mass/RV volume ratio of >0.3 g/ml) was further found to be a risk factor for SCD. Other risk factors assessed by this trial found to have an association with SCD were reduced LV and RVEF and a history of sustained atrial arrhythmia (59).

Cardiovascular magnetic resonance ventricular function parameters are a useful risk stratification tool for patients with Eisenmenger’s syndrome. A study by Jensen et al. found that CMR derived RVEF less than 40% and LVEF less than 50% was associated with increased risk of death in Eisenmenger’s patients with post-tricuspid shunts (60).

## Myocardial Fibrosis

Myocardial fibrosis may be a final common pathophysiological pathway that links a wide spectrum of congenital heart conditions. There is great importance in detecting its various forms and understanding its prognostic significance for a more targeted treatment approach. Broadly, there are two forms of fibrotic processes that can occur: replacement fibrosis and interstitial fibrosis.

Replacement fibrosis is irreversible and occurs following an insult to myocytes, commonly ischemia. This focal type of fibrosis can be detected usually by LGE imaging, assuming there is a neighboring normal myocardium.

Interstitial fibrosis is secondary to increased collagen deposition within the extracellular matrix (ECM) as a response to abnormal loading conditions on the myocardium such as typically occurs in CHD patients. It can be detected with T1 mapping and extracellular (ECV) measurements and remains undetected by LGE imaging because it is more diffuse and widespread throughout the myocardium preventing identification by comparison to neighboring “normal” myocardium.

Technical developments in LGE imaging, T1 mapping, and ECV measurements have enabled the non-invasive quantification of myocardial fibrosis that was previously only possible on a biopsy or during postmortem. A recent study has demonstrated that the incremental value of detecting focal fibrosis using LGE alongside conventional parameter (LVEF), in predicting 5-year mortality in patients with aortic stenosis (61). Furthermore, Halliday et al. have reported recently the value of mid-wall focal fibrosis as detected by LGE in predicting SCD in patients with dilated cardiomyopathy and LVEF >40% (62).

Diffuse myocardial fibrosis or interstitial disease is increasingly of interest. Its presence leads to abnormal T1 (pre- and postcontrast agent) and abnormal ECV measurements, which is speculated to predate cardiac dysfunction. Previous studies of cardiomyopathy have demonstrated correlation between increased ECV with diastolic dysfunction (63) and reduced myocardial blood flow (64). Correlation was found between ECV and reduced ventricular EF in a study of 50 patients with adult CHD (65).

## Focal Fibrosis and Scarring

### Late Gadolinium Enhancement

The differences between normal and abnormal myocardial tissue may be subtle on CMR; however, differences between these tissues are emphasized after the administration of intravenous gadolinium-based contrast agent (GBCA). Gadolinium, in its chelated form, is rendered non-toxic, and its distribution is confined to the extracellular compartment due to its large molecular weight. The proportion of extracellular space in a healthy myocardium is ~15% and increases in with heart disease due to increased collagen deposition and fibrosis. This results in an increased volume of distribution of gadolinium that further is compounded in conditions where the myocardial function is depressed; slowing down the gadolinium washout kinetics. Gadolinium has paramagnetic properties that allow it to interact with spins promoting more rapid exchange of energy thus T1 is shorter and results in a high signal (bright).

In practice, 0.5 μg to 0.1 μg/kg bolus GBCA is injected, and after 10–30 min, images are acquired. An inversion recovery fast gradient echo sequence is used in breath-hold. LGE imaging is performed using a non-selective 180° inversion preparation pulse. Following the inversion pulse, the longitudinal magnetization returns to its original value exponentially with a time constant T1. Due to the difference in GBCA concentrations between normal and abnormal myocardium, there is a difference in T1 values and hence different magnetization recovery curves. The inversion time (Ti) of normal myocardium is the time after the inversion pulse when the magnetization of normal myocardium is passing through 0 (the null point). At this Ti, normal myocardium is black in the resulting images. This Ti varies with GBCA dose, time after administration, and with patient-specific factors such as kidney function and disease state. In order to maximize the contrast between abnormal and normal myocardium, normal myocardium should be nulled. The Ti can be determined empirically or by performing a breath-hold Ti scout acquisition (using a Look–Locker sequence). This is either calculated manually or by performing a Ti scout (Look–Locker sequence). The Ti scout sequence acquires images at multiple time points following a single-inversion recovery pulse. Each image is therefore effectively acquired with a different Ti, and visual inspection allows the Ti to null the signal from normal myocardium to be determined. The optimal Ti needs to be increased as the study proceeds as the GBCA washes out of the myocardium. The inversion pulse and data acquisition are repeated on every second R–R interval to allow full T1 relaxation between pulses, which improves the SNR and makes the sequence less sensitive to variability in the R–R interval. In cases of tachycardia, images can be acquired on every third R–R interval at the expense of increasing the imaging time. With conventional LGE imaging, incorrect setting of the Ti adversely affects the contrast between normal and abnormal myocardium.

Common challenges in patients with CHD are difficulties of ECG gating due to arrhythmia, wide and abnormally shaped QRS complexes, breath-holding difficulties as well as metallic artifacts from sternal wires and transcatheter devices. LGE false positive scans occur more commonly when imaging the thin-walled RV due to partial volume effects. Partial volume effects are seen when one pixel contains both normal tissue and abnormal myocardium (e.g., fat and RV wall). Partial volume effects can be reduced by acquiring images with higher spatial resolution and in a shorter acquisition window (to reduce motion blurring) but both of these will increase breath-hold duration. Acquiring images in systole (when the RV wall is thickest) can be beneficial. In patients who have intracardiac shunts, the washout kinetics of gadolinium are different and hard to predict as GBCA clearance can be rapid (66).

To reduce false positive diagnosis of LGE phase swapping, cross cutting and comparing the LGE images with cine images are helpful. Imaging the RV for small areas of fibrosis requires meticulous choices in Ti.

While conventional LGE images are magnitude reconstructions that take no account of the polarity of the magnetization following the inversion pulse, phase sensitive inversion recovery (PSIR) LGE image reconstructions take into account both the magnitude and the polarity. With this type of reconstruction, the sequence is more tolerant to incorrect setting of the Ti to null normal myocardium, and the contrast between normal and abnormal myocardium is maintained over a range of values (67). While the inversion pulses are output and the data segments acquired on alternate cardiac cycles, with the PSIR sequence, additional low flip angle data are acquired in the redundant cardiac cycles and used to correct for other sources of phase variation in the reconstruction.

In cases where patients may find breath-holding difficult, motion corrected, free breathing, PSIR LGE imaging helps (68). In this technique, complete images are acquired using an inversion prepared bSSFP in each of a number of cardiac cycles. Non-rigid motion correction of these low SNR images is then performed (to correct for respiration) prior to averaging to generate a single high-SNR image. This technique has been shown to produce high quality images in patients with poor breath-hold capability and with arrhythmia (68). We have found this to be an excellent approach in CHD including for the RV.

For 3D coverage of the heart with high spatial resolution, respiratory-gated free breathing acquisitions can be performed. These navigated sequences may enable the assessment of thin walled structures such as atria and RV free wall although acquisition durations are long (typically 5–10 min) and unpredictable (69).

### Clinical Applications of LGE Imaging

#### Repaired Tetralogy of Fallot

**Figure 2:**
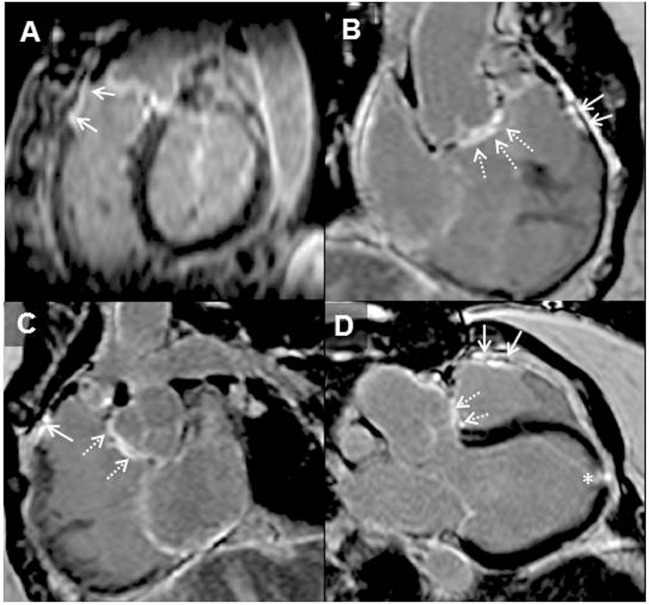
**Adult patient after repair of tetralogy of Fallot and later surgery for pulmonary valve implantation**. Solid arrows demonstrating late gadolinium enhancement (LGE) in the right ventricle (RV) outflow tract **(A–D)**. Broken arrows showing LGE in the ventricular septal defect region **(B–D)**. LGE of the apical vent site following surgery* **(D)** is also present. Where present, these may be useful when acquiring LGE images to guide optimal nulling of the normal myocardium; RV–left ventricle inferior insertion point faint LGE is ubiquitous **(A)** and similarly useful (66).

Patients with *repaired ToF* encounter problems in adulthood relating to RV dysfunction, pulmonary regurgitation, and clinical arrhythmia and are at greater risk of SCD. Detection of myocardial scarring has contributed to the understanding of RV dysfunction late after repair. We systematically investigated LGE and clinical status in a cohort of adult ToF patients and found focal areas of fibrosis not only at the ventriculotomy, VSD repair, and apical vent sites but also in remote locations in the LV and RV wall and trabeculations (12) The RVOT was an important area of CMR defined focal fibrosis and dyskinesia (see Figure 2). Though by definition of the surgical intervention required to repair the heart the RV always has LGE in this region, the extent is highly variable.

There are several postulated mechanisms that explain the distribution of focal fibrosis seen in repaired ToF. Fibrosis in remote areas of the LV and RV could be as a result of ischemic insult in the pre-, peri-, or postoperative phase or related to RV hypertrophy and dilation secondary to pulmonary stenosis or regurgitation. Older patients with repaired ToF were reported to have greater LGE in the RVOT and dyskinesia probably relating to previous surgical methods of reconstructing the RVOT such as transannular patch repair that involved extensive resection (12). The amount of LGE found in the RV was associated with RV dysfunction, exercise intolerance, and previous presentation with clinical arrhythmia (12). In a separate study including heterogenous operated and unoperated CHD patients, the absence of LGE together with good peak oxygen consumption on exercise testing was found to correlate with lack of inducible ventricular tachycardia (70). Other groups revealed that RV fibrosis is associated with diastolic dysfunction and surface ECG abnormalities associated with arrhythmia in ToF patients (71, 72).

#### Systemic RV after Atrial Redirection Surgery for TGA

In patients with *transposition of TGA* who underwent *surgical palliation by atrial redirection surgery*, the RV needs to sustain the systemic circulation for the long term. The hemodynamic burden on the maladapted systemic RV can lead to RV dysfunction, reduced exercise capacity, arrhythmia, and SCD. A likely mechanism of dysfunction of the systemic RV is a myocardial perfusion mismatch leading to myocardial ischemia and fibrosis (12).

**Figure 3:**
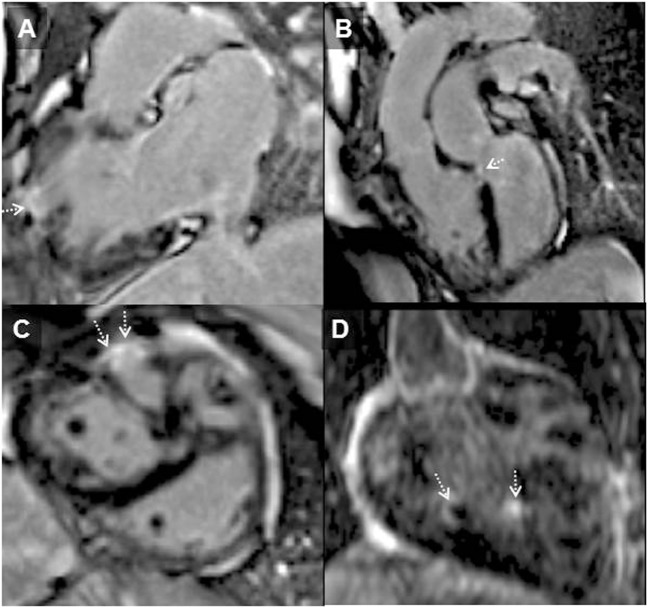
**Systemic right ventricle (RV) following atrial redirection surgery (Mustard operation)**. RV in-out view **(A)** showing free wall localized transmural RV late gadolinium enhancement (arrowhead); LGE in the ventricular septum consistent with previous VSD repair [**(B)**; dotted arrow], in the free wall of the RV [**(C)**; dotted arrows] and of trabeculations within the body of the RV [**(D)**; dotted arrows]. This is the typical pattern of fibrosis seen in systemic RV following atrial redirection surgery (76).

**Figure 4:**
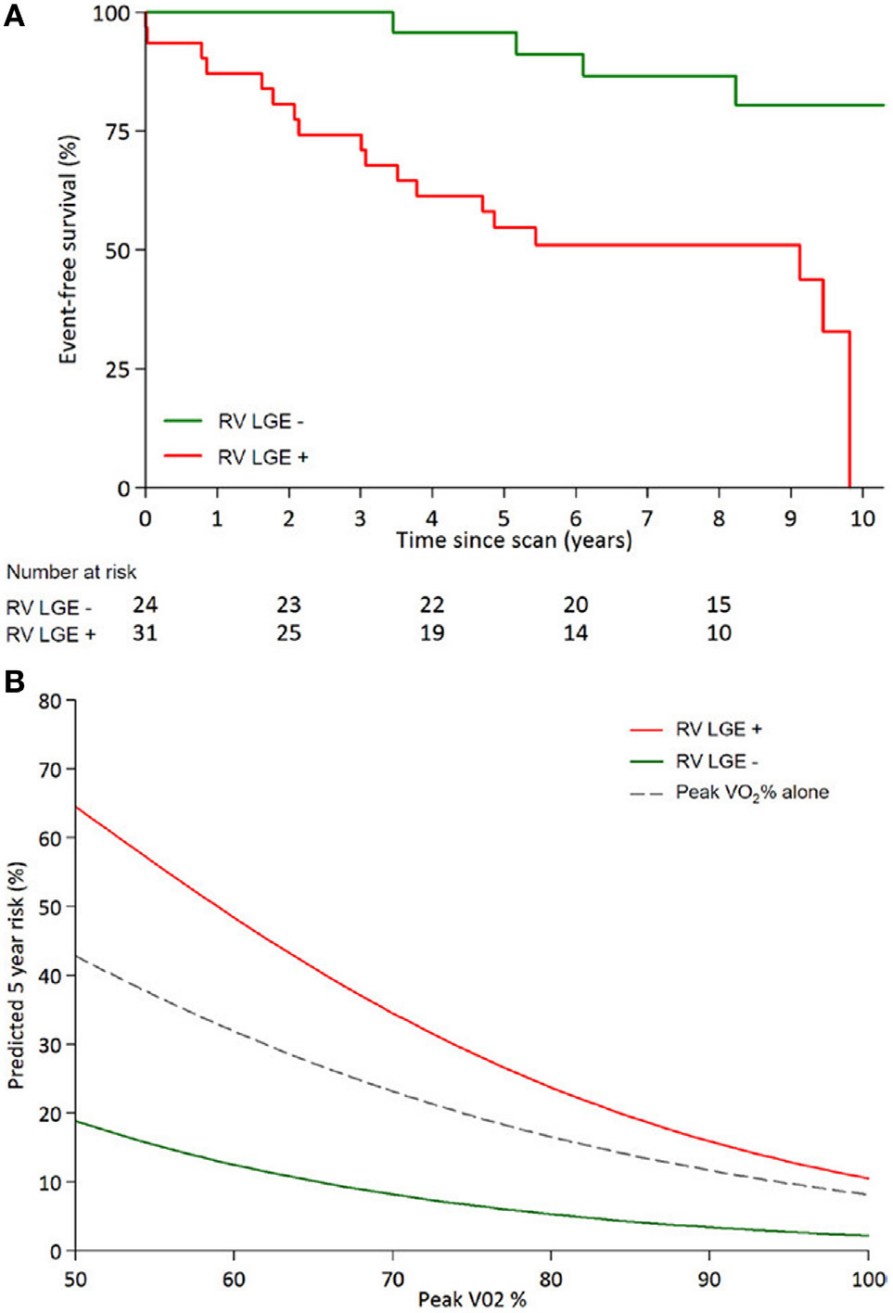
**Kaplan–Meier curves adapted from Rydman et al. (A)** Event free survival from combined clinical composite end points (arrhythmia, congestive heart failure, transplant, and death) for systemic ventricles post-atrial redirection surgery showing a significant difference between patients with fibrosis [late gadolinium enhancement (LGE)] and without fibrosis (Log-rank *P* = 0.001). **(B)** Fibrosis (LGE) contributes significantly to prediction of 5-year survival when used in conjunction with predicted peak VO_2_ (peak VO_2_%), compared to peak VO_2_ alone (73).

The first prospective study to investigate myocardial fibrosis by LGE imaging in individuals with systemic RV following atrial redirection surgery found that RV LGE was present in 56% of studied patients. Among other variables in the study, the mere presence of RV LGE independently and strongly predicted adverse clinical outcomes (atrial/ventricular arrhythmia and death) with a hazard ration of 4.95 (73) (see Figures 3 and 4). The extent of LGE correlated with age, RV dysfunction, and dyssynchrony as well as clinical arrhythmia (73, 74). Focal fibrosis has also been demonstrated also by others in the systemic RV (congenitally corrected TGA and Mustard/Senning) correlating with RV dysfunction, arrhythmia presentation, and exercise intolerance (75).

Late gadolinium enhancement was also studied in patients who underwent the *arterial switch operation for TGA*, a population at risk for coronary stenosis, occlusions, and late death. Despite the reported prevalence of 3–7% for coronary problems, LGE was only found in 1.8% of 220 patients. Approximately 20% of patients had (mostly mild) LV dysfunction post-arterial switch surgery (77, 78).

#### Single Ventricle

**Figure 5:**
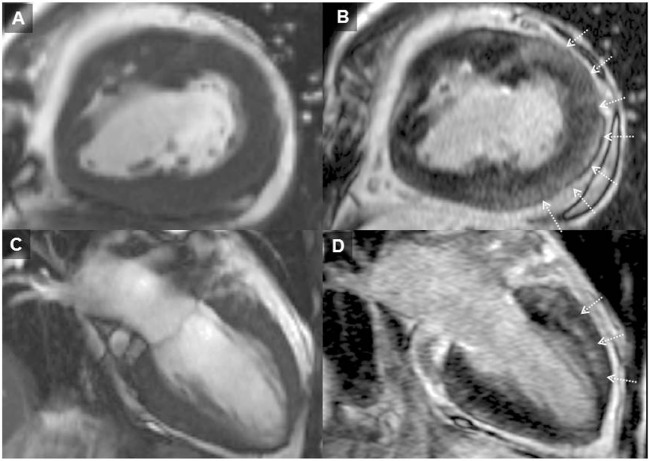
**Cardiovascular magnetic resonance images status post-lateral tunnel total cavopulmonary connection surgery for single-left ventricle (LV) physiology**. **(A)** and **(C)** (2D cine images) compared to **(B)** and **(D)** (LGE images). Arrows represent diffuse appearance late gadolinium enhancement in the free wall of the LV, which is the primary ventricle. This kind of fibrosis pattern in Fontan is one of the several previously described patterns (82).

While undergoing several surgical procedures in the first years of life, *single-ventricle* patients are affected by significant morbidity and mortality (79, 80). Ventricular dimensions and dysfunction played a major role in the long-term outcome in a cohort of 215 patients after the Fontan palliation (81). In particular, the end-diastolic volume of the primary ventricle was the strongest volumetric parameter associated with adverse clinical outcomes (81). Myocardial fibrosis as imaged by LGE was detected in 28% of patients studied. Although LGE was found at the surgical sites as expected, this was only in 8% of cases. The large majority of the LGE distribution was found in the free wall of the primary ventricle (64%) with lesser degrees in the secondary ventricle (36%), septal insertion points (16%), papillary muscles (12%), and ventricular apex (8%) (82). LGE lesions were categorized as transmural (40%), subendocardial (36%), and diffuse LGE (12%) (see Figure 5). The presence and extent of ventricular LGE closely correlated with adverse ventricular size and function as well as non-sustained ventricular tachycardia, all risk factors for poor clinical outcomes (82).

#### Eisenmenger Syndrome

A small study in *Eisenmenger* syndrome involving 30 patients showed that a large majority had LGE (73%), of which 70% was found in the RV myocardium but LV LGE was also found. No correlation with ventricular size and function, exercise capacity, or survival was found. Fast gadolinium washout kinetics was also found (66). Therefore, to date, the routine use of LGE for this condition was not considered justified (83).

## Diffuse and Interstitial Fibrosis

### T1 Mapping

Diffuse myocardial fibrosis has been shown to be an important predictor for SCD, ventricular tachycardia, and heart failure. Rapid advances in CMR have enabled the non-invasive detection and quantification of diffuse myocardial fibrosis and interstitial disease in the myocardium using T1 mapping (84, 85).

After the magnetization has been excited with a radio-frequency pulse, it relaxes back to its equilibrium state in the longitudinal direction aligned with the main magnetic field. T1 is a property constant characterizing how long the magnetization takes to recover to 63% of its original longitudinal equilibrium value in the main field. The rate of T1 relaxation is dependent on each proton’s ability to exchange energy with its surroundings. The T1 constant differs between tissues depending largely but not solely on the concentration of water. The paramagnetic properties of (ionic) iron and (complex chelated ionic) gadolinium shorten T1. With T1 mapping, an estimate is calculated from a series of different T1-weighted images during the magnetization recovery process and is written as a “T1 map” where the T1 value of each pixel is encoded in to the intensity of the T1-map pixels. This involves a series of assumptions about alignment of the pixels in all images of the supporting series (typically subject to errors such as inter-cycle differences, breath-hold imperfection, and corruption by blood signal artifacts) (86, 87).

### T1-Mapping Imaging Sequences: Modified Look–Locker Imaging (MOLLI)

Following a 180° inversion pulse, single-shot images are acquired in typically diastole, typically over three to five heart beats at different inversion recovery times (Ti). After a recovery period to allow for complete T1 recovery another, inversion pulse is applied followed by a similar series of images but slightly different Ti values thereby sampling more points along the T1 recovery curve. Images are then organized in order of increasing Ti (88). When this process is performed for all pixels in the image, a T1 map is created. MOLLI acquisition duration is heart rate dependent; the greater the number of beats involved in the acquisition, the longer the breath-hold. Therefore, a shortened MOLLI sequence is often used acquiring single shots during five heart beats, followed by three beats recovery, and then a second “set” of single shots acquired over three beats following a second inversion pulse, written as 5(3)3 (89). This allows for a breath-hold T1 map taking only over 11 beats rather than the 17 beats in the original innovative sequences used. A modified arrangement 4(1)3(1)2 is applied postcontrast because its arrangement of TI values is more suitable to the shorter T1 values post-gadolinium (89).

### Shortened Modified Look–Locker Imaging (shMOLLI)

An “shMOLLI” is a shorter sequence that involves image acquisition during fewer heart beats thereby shortening breath-hold time (90). An example is acquiring single-shot images over the first five beats followed by one recovery beat and cycles thereafter that are only single beat in duration 5 (1)1(1)1. This reduces the breath-hold time to nine beats; however, this approach is suited best to long-native T1 values rather than the short-post-Gad values.

### Saturation Recovery Single-Shot Acquisition (SASHA)

Following an initial acquisition of an image taken with the magnetization vector being in the equilibrium state, a saturation recovery pulse is used multiple times instead of an inversion pulse. At varies times from the application of the saturation pulse (Ts), images are acquired. The signal intensity of the pixels is fitted as a function of the Ts values acquired to calculate the pixel-wise T1 map. The SASHA technique is more accurate at estimating T1 whereas MOLLI and shMOLLI may underestimate T1 with various reasons for their bias; however, SASHA is less precise mainly because it uses of a saturation pulse compared to an inversion pulse leading to reduced “contrast-to-noise” in the fitting process and therefore more random noise in the T1 maps. An advantage is that it is less susceptible to changes in R–R intervals and therefore may perform better in arrhythmias. Some debate continues over the optimum T1-mapping approach for maximum sensitivity to disease (while accuracy of T1 itself is possibly less important except that leads to difficulties in widespread clinical inter-vendor multicentre use), while also having minimal scatter (imprecision) for technical factors: these aspects are to a large extent contradictory in the physics requiring further development for early detection of diffuse fibrosis as no adequate solution seems evident.

There are two main techniques involving T1 mapping: native T1 is a non-contrast technique that detects pathological changes in the myocardium that occur associated with a relative increase in the concentration of water (edema) or increased interstitial space caused by protein deposition (from increased collagen causing fibrosis) or other proteins such as amyloid. The increase in native T1 is not specific to diffuse fibrosis because it occurs also (and somewhat more strongly) in other disease processes such as myocarditis and infarction. The advantage is that it can be used in patients with severe renal failure as it does not involve contrast agent. Crucially, it avoids contrast agent in CHD patients who are likely to require multiple follow-up scans from a young age (91).

### ECV Measurements

This requires the application of extracellular GBCA. The volume of the myocardium that is not taken up by cardiomyocytes is the extracellular compartment, which contains the ECM. ECV is not identical to the ECM, which cannot be measured. However, ECV is a surrogate marker that is to some unclear extent sensitive to an abnormal amount of ECM.

ECV is derived by measuring the change in tissue T1-relaxation rate (R1 = 1/T1) and comparing this to the change in blood T1-relaxation rate pre- and postcontrast application, built on several assumptions, also requiring knowledge of the hematocrit (88). The more fibrosis in the extracellular space, the longer the native T1 because from a physics perspective, the average water content of the myocardium is slightly higher with more interstitial fluid (intracellular cytoplasm containing a high concentration of macromolecules). However, on the images postcontrast, there will be more GBCA accumulation in the increased interstitial space of abnormal myocardium, therefore shortening post-gadolinium T1 relative to normal myocardium. The changes in 1/T1 for tissue, when used in the formula to calculate ECV, result in a larger ECV value. In normal subjects, gender-related associations of ECV with age have been demonstrated, with some contradictions (92).

In the absence of edema, the pathological expansion of the ECM is primarily due to the increased proportion of myocardial collagen within the matrix. This fibrotic process leads to mechanical, electrical, and vasomotor dysfunction. Investigators have reported the interstitial disease association with SCD (84, 85, 93). This ECM expansion may have important implications for identifying distinct therapeutic targets.

### Clinical Applications of T1 Mapping

Clinical studies reported to date mostly include the systemic LV. The RV is much thinner. For the RV myocardium, unless it is systemic or for some other reason abnormally thickened or immobile, the T1-mapping methods face major technical limitations affecting reliability, such as adjoining fat and blood signals next to the thin trabeculated highly mobile RV wall. Suppression of these corrupting signals is usually associated with some additional unreliability or loss of myocardial data, further complicated by the proximity of sternal wires, and new MRI methods are needed to make RV T1 or ECV clinically reliable (94). The difficulties that are encountered with T1 mapping and ECV become less important in the diagnosis of conditions such as cardiac amyloid and Anderson Fabry’s disease where there large abnormalities in the T1 and ECV indices; however, they are affected when this technique is used to detect and monitor subtle changes in diffuse fibrosis.

The presence of interstitial fibrosis has been shown to be of prognostic significance in the general adult cardiology population. In the CHD population, we can speculate that non-invasive detection of interstitial fibrosis may offer better tracking of RV disease than currently utilized volumes and ejection fraction. However, even in those patients with CHD and relatively thicker RV such as systemic RV after atrial redirection surgery, HLHS, ToF, and CHD patients with pulmonary hypertension but extrapolation of current techniques for the LV cannot be assumed.

The only prospective study of T1 mapping and outcomes in tetralogy of Fallot relates to LV ECV in the study by Broberg and colleagues. Approximately 25% of subjects with rToF had elevated LV ECV compared to the control group, of which subjects with LV ECV >30% had significant clinical events (sustained clinically relevant arrhythmia and death) during their follow-up (65). More patients with myocardial fibrosis were identified by LV ECV than with LGE, consistent with other studies (95). LV ECV was abnormal in patients with normal LV ejection fraction suggesting that interstitial fibrosis could precede systolic dysfunction and therefore have a potential role as an early biomarker for myocardial disease (65).

In a different study, 11 patients with a systemic RV had higher ECV, which correlated with elevated end-diastolic volumes and impaired ejection fraction (95). A further study in patients with systemic RV found high ECV measurements in the interventricular septum (the free RV wall could not be reliably studied), which correlated with higher B type natriuretic peptide levels (96).

Several studies have investigated biventricular diffuse fibrosis in ToF subjects using T1 mapping and ECV. However, these were cross-sectional studies with limitations in their methodology, namely, using postcontrast T1 times only due to unavailable hematocrit values (technique not validated to measure diffuse fibrosis in the RV) (97) and limited methods for optimizing MRI sequences or acquisition planes to mitigate challenges of RV imaging (97, 98). High RV ECV was associated with lower RVOT pressure gradient and lower RV mass-to-volume-ratio. LV ECV was found to correlate with arrhythmia, but this category included frequent ventricular systoles (>100 beats or more in 24 h), and the relevance to clinically significant arrhythmia in ToF needs further study (98).

Native T1 times and ECV in LV myocardium were measured in 31 rToF patients and compared to controls. Prolonged cardiopulmonary bypass and aortic cross-clamp time during their previous surgery, biventricular enlargement, and reduced exercise tolerance correlated with higher native T1 times and ECV in the LV (99).

## Further Challenges

From the interrogation of the intricate myocardial architecture and its properties, to assessment of regional and global myocardial function, cardiac MRI has proven invaluable in providing data that have several important clinical implications for CHD patients.

The functional assessment of the RV is very important for CHD patients as it is the chamber that is most often affected, yet it is also the chamber that is most challenging to image due to its thin, highly mobile wall, and more complex geometry. The RV wall location may change between cardiac cycles due to inter-cycle variations in returning venous flow. Imaging of the RV can be further complicated by the proximity of postsurgical sternal wires. These factors make RV imaging an extreme technical challenge for the reliability of conventional cardiac MRI. On the other hand, as the RV is adjacent to the anterior surface coil in cardiac MRI, it can be imaged with high SNR.

A technique that shows potential in the study of the RV is black-blood LGE imaging. LGE imaging with the addition of PSIR is excellent in differentiating myocardial scar from normal myocardium; however, the contrast between subendocardial scar and blood pool on the white-blood images may be reduced and therefore scar may be difficult to identify. Furthermore, differentiation of fibrosis from fat and metallic artifact can be difficult. Black-blood LGE images using an inversion recovery T2 weighted SSFP have been proposed as one possible solution to this (100). Recently, Kellman and colleagues showed good results using this sequence in patients with subendocardial myocardial infarction. They speculated that black-blood LGE sequences may help imaging thin walled fibrous structures (100).

Quantification of diffuse fibrosis with T1 mapping until now has largely been for research interest rather than clinical application. Its clinical value in CHD patients still requires clarification. Technical challenges that lie ahead include the difficulty of mapping the relatively thin-structured RV walls. Furthermore, the potential role for T1 mapping and ECV to help identify and target treatment for “reversible” interstitial disease remains unclear. A study investigated diffuse fibrosis post-aortic valve replacement for aortic stenosis with T1 mapping and found that diffuse fibrosis persistent at 6 months following aortic valve replacements despite normalization of LV-loading conditions and regression of LVH (101), suggesting that interstitial fibrosis may not be a reversible process after all. Second, in the presence of late gadolinium representing scar, the additional prognostic value of diffuse fibrosis measurement remains unclear. If T1 mapping and ECV prognostic capability becomes well established as new biomarkers for CHD, this can open new investigational avenues to develop targeted therapeutic interventions that can retard the fibrotic process.

Cardiac DTI may have potential future use in identifying intrinsic differences in myo-architecture that predates fibrosis associated with remodeling. With further development, cDTI may provide non-invasive means of identifying areas of myocardial disarray as potential substrates (anatomical isthmuses) for arrhythmia propagation, which can help guide electrophysiological therapeutic intervention. Further development, however, is required to improve the spatial resolution of cDTI including to enable it to differentiate myocardial fiber orientation in the relatively thinned walled RV (a chamber commonly of clinical interest in CHD), and therefore its use today is limited to that of a research tool.

## Author Contributions

SB-N and SG were responsible for the conception and design of the work. SB-N and IV substantially contributed to CMR and CHD content. MG substantially contributed toward CHD content. PG and JK substantially contributed to MRI physics content. PK substantially contributed to the myocardial mechanics and myocardial architecture section. SB-N and SG contributed to the first draft and revisions. All authors contributed to revising the work critically for important intellectual content prior to final approval. The final approval of the manuscript was done by SB-N.

## Conflict of Interest Statement

The authors declare that the research was conducted in the absence of any commercial or financial relationships that could be construed as a potential conflict of interest.
